# Evaluating Distal and Proximal Explanations for Withdrawal: A Rejoinder to Varnum and Kwon’s “The Ecology of Withdrawal”

**DOI:** 10.3389/fpsyg.2017.02085

**Published:** 2017-11-29

**Authors:** Vinai Norasakkunkit, Yukiko Uchida, Kosuke Takemura

**Affiliations:** ^1^Department of Psychology, Gonzaga University, Spokane, WA, United States; ^2^Kokoro Research Center, Kyoto University, Kyoto, Japan; ^3^Faculty of Economics, Shiga University, Hikone, Japan

**Keywords:** withdrawal, *NEET*, *Hikikomori*, marginalization, youth, Japan, globalization, evolutionary psychology

## Abstract

In their 2016 commentary on our theorizing about how youth withdrawal from economic and social participation in Japanese society (i.e., *NEET* and *Hikikomori* phenomena) stems from generational inequality of economic opportunities, Varnum and Kwon correctly point out that our explanation for withdrawal is yet untested. They then offered an alternative, evolutionary psychological explanation for withdrawal in which they claim that in resource-rich ecologies like Japan, the option to withdraw from participating in society is a possible life strategy, a strategy that would be much more costly in resource-poor ecologies. While we agree with this premise, we argue that this distal explanatory framework, at least in its current form, has limits in reconciling some of the more recent cross-cultural observations, as well as well-established sociological claims about the causes of withdrawal. Thus we argue that much work remains in refining and expanding the explanatory power of more distal explanations on the issue of withdrawal. Until then, the more proximal and culture-specific explanations are probably the useful and meaningful explanations for the withdrawal phenomenon.

## Background

According to Japan’s Ministry of Health, Labor and Welfare those who withdraw from occupational participation are called *NEET* (i.e., *Not* in *Employment, Education*, or *Training*), and those who withdraw from social participation for 6 months or longer are called *Hikikomori* (i.e., a social isolate or recluse). Both *NEET* and *Hikikomori* youth in Japan represent a large enough proportion of the Japanese youth population that these phenomena are regarded as a concerning social problem by the Japanese government.

In our previous empirical work published in this *journal* (Uchida and Norasakkunkit, 2015) and elsewhere (Norasakkunkit and Uchida, 2011, 2014), we have treated *NEET* and *Hikikomori* as a spectrum (henceforth referred to as the N/H Spectrum) and have also developed a scale called the *N/H Risk Scale* or *NHR Scale* to measure where an individual lies on this spectrum. In our 2015 publication, we provided evidence for the predictive validity of this scale by showing that higher risk scores on this scale were incrementally associated with greater degrees of occupational marginalization, as well as lower levels of educational attainment, in a nationwide sample in Japan.

With respect to psychological tendencies associated with N/H Risk, our experimental work found that high scores on the NHR scale are associated with culturally deviant motivational styles (Norasakkunkit and Uchida, 2011, 2014). Specifically we found that *low* N/H risk Japanese participants behaved as mainstream Japanese do in: (1) being more motivated to persist on a difficult task when they received negative feedback than when they received positive feedback (see Heine et al., 2001), and (2) being more motivated to comply to a request for the sake of maintaining social conformity than for the sake of maintaining self-consistency (see Cialdini et al., 1999). In contrast, we found the reversed patterns in these motives for the *high* N/H risk Japanese participants. Furthermore, we found that these differences in these motivational styles between high and low N/H risk Japanese participants were mediated by levels of personal endorsement of culturally dominant interdependent values. A more recent empirical study has also confirmed similar patterns of association between NHR scores and culturally deviant behavioral tendencies (Ishii and Uchida, 2016).

Our theoretical work published in this journal (Toivonen et al., 2011) and elsewhere (Norasakkunkit and Uchida, 2012; Norasakkunkit et al., 2012) have argued that the *reasons* for the culturally deviant motivational styles and values among occupationally and/or socially withdrawn Japanese youth stem from their relative lack of access to equal opportunities to enter the core of Japanese labor markets. This is due to the persistence of uncompetitive institutional practices like the seniority system, as well as the breakdown of past mechanisms that smoothly transitioned youth from school to the workplace (for a discussion on this, see Toivonen et al., 2011). Consequently, many Japanese youth are shut out from the opportunity to secure a place of belonging in the mainstream of society. This position of marginalization presents a lack of incentive to fully internalize mainstream cultural values and norms while, at the same time, not having access to an alternative value system to internalize in its place. In other words, economic marginalization leads to demotivation, deidentification, and withdrawal, not the other way around.

## Response to Our Work

Varnum and Kwon (2016) provided an insightful commentary titled, *The Ecology of Withdrawal*, on our work summarized above. In it, Varnum and Kwon correctly highlighted that any cause of the N/H phenomenon discussed in our past articles have been speculative and that the actual cause of N/H remains unknown. They then proceeded to offer an alternative explanation from an evolutionary psychological perspective, suggesting that: (1) social and occupational withdrawal as a life strategy is probably only possible in resource-rich sociocultural ecologies, like those of Japan and the United States, or else such a strategy would be too much of a threat to survival, and (2) adopting a version of Life History Theory (Del Giudice et al., 2015), the authors suggested that those with dispositions toward *fast life history strategies* (i.e., “preference for immediate rewards, impulsivity, aggression, early and more frequent reproduction”) would be less adapted in resource-rich post-industrial sociocultural ecologies where a *slow life history strategy* (i.e., “greater investment in long-term outcomes, delayed reproduction”) would be more adaptive. The incompatibility between the dispositions toward fast life history strategy and an ecology where a slow life history strategy would be more adaptive may thereby lead to a life strategy of social and occupational withdrawal as a manifestation of the misfit between disposition and ecology.

While the authors admit that, at first glance, it seems puzzling that a fast life history strategy could manifest as “withdrawal” rather than as acting out behaviors (e.g., criminal behaviors, aggression, fast and more frequent reproduction) they proposed that withdrawal can become an option *only* in a resource-rich ecologies because it is only in such ecologies that withdrawal from social and occupational life would not necessarily be a threat to survival.

## Our Response to Varnum and Kwon’s Commentary

We greatly appreciate the proposed causal explanation that Varnum and Kwon have offered, as it is always important to consider more distal explanations as part of the story of even more recent psychological phenomena. Indeed, such distal explanations are well suited to explain macro-level differences, such as the relationship between GDP and prevalence rates of *NEETs* at the country level. Resource-rich societies may make withdrawal a possible option as a life strategy, at least for some individuals. Thus, we whole-heartedly agree that withdrawal would be more challenging to sustain in resource-poor ecologies. Furthermore, just like with Hofstede’s approach (Hofstede, 1980), it is possible to check how macro level cultural norms, such as individualism or collectivism, might promote certain behavioral strategies.

However, distal explanation can have limited power in explaining micro-level individual differences within a culture. For example, in our previous studies (see Norasakkunkit and Uchida, 2011, 2014), we have found that high NHR scorers, relative to low NHR scorers, tend to have personal values that are deviant from those that are considered normative in Japan. Yet, harboring culturally deviant values may not be compelled by personal choice alone, as it can also be compelled by marginalizing circumstances such as inequality of economic opportunities.

Varnum and Kwon discuss how withdrawal in resource-rich ecologies occurs for those who have “failed at using slow strategies” (p. 2). We are not clear here if Varnum and Kwon are necessarily suggesting that this “failed strategy” is a result of primarily individual disposition. However, we think that the claim here can very easily sound like the individual is to blame for his/her own withdrawal. While we are certainly open to thinking about the role that individual choices play in withdrawal, we believe that it is important to rule out primarily situational causes first. Indeed, the dispositional causal framework would challenge much of sociological discourse that has been highlighting unequal economic opportunities as the major cause of social and occupational withdrawal for quite some time (e.g., Saito, 1998; Genda, 2005; Zielenziger, 2006; Kosugi, 2008; Slater, 2010; Brinton, 2011; Yamada, 2011; Allison, 2013). Therefore, explaining the N/H phenomenon only through the lens of life strategies may sound too much like victim blaming that supports a just-world hypothesis (i.e., people get what they deserve and deserve what they get; Lerner, 1980). From this framework, it is not too far of a leap to suggest that poor people are never poor because of their circumstances but because of a deficit in their internal capacity to climb out of poverty-stricken conditions.

Consequently, if Varnum and Kwon are essentially suggesting that withdrawal is largely a function of failing to adapt to resource-rich ecologies, we believe this framework may be over-individualizing the problem by overestimating the role that individual choices play. We believe this view would undermine and decontextualize the complexity of the problem in question.

Like the sociologists and anthropologists cited above who have studied the withdrawal phenomenon for nearly two decades by now, we also believe it is possible for those who would otherwise be adapting successfully to resource-rich ecologies to withdraw as a result of being in underprivileged socioeconomic circumstances. Part of the relevant ecology here is not only that it is a resource-rich ecology but also that there is inequality of opportunities for everyone.

To take an example in the United States, a very innately intelligent person born in a poor neighborhood is likely to be cut off from the legitimate means to achieving life success (see Putnam, 2015). These circumstances are painfully obvious to the individual in question, which itself can be quite demotivating with respect to adopting the ecologically adaptive life strategy. Indeed, a recent cross-national meta-analytic study on gene-SES interactions suggests that this seems to be the case in the contemporary American cultural context. Inequality of opportunities is becoming an ever-increasing reality such that no matter how much genetic predisposition to intelligence one has, the socioeconomic circumstances into which one is born much more likely determines actual academic success (Tucker-Drob and Bates, 2016).

It is also noteworthy to point out, as mentioned in the original article, that we found a strong negative correlation between *NEET*/*Hikikomori* Spectrum scores and SES in Japan. In fact, more recently, we found this to be the case in the United States as well (Norasakkunkit et al., 2015, 2016a). Thus, even though those who tend to score high on the NHR scale may be living in a resource-rich national ecology, their family resources are usually relatively limited. Furthermore, it is not clear how much access low SES individuals have to their society’s social safety net to enable their tendency to withdraw. At least in Japan, public aid is only available if self-help and family support are not available at all (Vij, 2007).

A limitation to Varnum and Kwon’s framework, at least as it applies to the Japanese cultural context, is represented by Inglehart et al.’s (2004) analyses of the World Values Survey and European Values Study (1999–2002). While they reported that rich countries like France, Great Britain, and Sweden tended to view work as less important in their lives, which is consistent with Varnum and Kwon’s claim that withdrawal from work would be more common in resource rich ecologies, one obvious exception was Japan. Though Japan is a rich country, only 5% of respondents endorsed the view that work was less important in their lives. While our own data (Uchida and Norasakkunkit, 2015) suggests that withdrawn people in Japan would be among those 5%, it is not clear how Varnum and Kwon’s framework would explain why this attitude among the withdrawn in Japan represents an exception in their own society, as opposed to simply reflecting a more extreme form of a general trend in society, which seems to be the case in other resource-rich ecologies.

One possible compromise approach between our more proximal framework and Varnum and Kwon’s more distal framework is to conduct a multi-level analysis while collecting individual-level data from multiple nations that represent a variety of socioeconomic conditions. At the individual level, individual experiences and perceptions that are associated with withdrawal behaviors (e.g., rejection of mainstream cultural values) can be measured. Then, at the macro level, we might find, as Varnum and Kwon suggests, that GDP explains the prevalence rates of social and occupational withdrawal across societies. Conducting this kind of multi-level analysis then makes it possible to see if there are interaction effects between macro level factors such as GDP and individual level factors. We may then find that in some rich societies, social and occupational withdrawal represents a strategy to rebel against mainstream values of that society while in others, withdrawal may represent something else, for example.

Thus, a distal explanatory framework for withdrawal is not necessarily incompatible with more proximal and culture-specific explanations, since these are different levels of explanations. However, more proximal and culture-specific explanations are free to vary across different contexts. In contrast, the distal explanation framework would have to address discrepancies across contexts in the data in a more overarching way. We believe this is where Varnum and Kown’s framework falls short. We discuss this in detail below.

First, it goes without saying that crime and other aggressive acts are abundant even in resource-rich ecologies such as the United States where homicide rates are seven times that of other high income countries, on average (Sumner et al., 2015). Thus, a distal explanatory framework would have to explain why there would be those who use the atypical fast life history strategy (i.e., withdrawal) while others use the more typical fast life history strategy (i.e., aggression, crime, more frequent reproduction). In other words, what distinguishes the individuals who would use one over another type of fast life history strategy in the same resource-rich ecology?

Second, when comparing high vs. low N/H risk across United States and Japan (e.g., Norasakkunkit et al., 2016b), it appears that the high risk group in Japan looks a bit like the low risk group in the United States with respect to motivational style. In contrast, this pattern is completely reversed in the United States such that the high risk groups in the United States looks a bit like the low risk group in Japan. Therefore, the distal explanation would have to explain why N/H tendencies are associated with culturally deviant motivational styles, and subsequently why the culturally deviant motivational style associated with withdrawal in one resource-rich ecology would look more normative in another resource-rich ecology. From our point of view, more than just the options that a resource-rich ecology would provide, there seems to be something about not being able to align oneself with predominant cultural norms in one’s society, whatever those norms are, and that this is a feature of the withdrawal tendency.

Finally, although we have not found gender differences in relative tendencies on our scale, the only systematically conducted nation-wide epidemiological study on *hikikomori*, arguably the most extreme form of withdrawal, suggests that *hikikomori* is most common among boys and men and relatively rare among girls and women (Koyama et al., 2010). Perhaps there is an evolutionary psychological explanation for this gender difference, but it is not clear how Varnum and Kwon’s explanation would account for this gender difference, assuming that this gender difference is a constant across resource-rich ecologies. In contrast, a proximal explanation only has to explain why the tendency to withdraw in the extreme form is more prevalent among Japanese men even if it were true that men are more prone to withdrawal than women across resource-rich ecologies. Nevertheless, Varnum and Kwon’s theory would certainly be more compelling if they can make a claim for why men may be more vulnerable to withdrawal than women and point to cross-cultural evidence for this claim.

## Conclusion

We think that the distal perspective still needs further theoretical work before it can reconcile the divergent observations that we discussed above in a satisfying and overarching way. In particular, we think the part about fast vs. slow life history strategy needs to be either further developed or discarded for the evolutionary framework to be useful in explaining the withdrawal phenomenon. Nevertheless, once a more compelling distal framework is available, we hope to be able to draw on such a framework to compliment our own culture-specific understanding of the N/H phenomenon. Until then, we will probably continue to draw primarily on our own proximal and culture-specific explanatory framework to understand the withdrawal phenomenon, at least in the Japanese cultural context.

## Author Contributions

VN was the primary commentator for this rejoinder to Varnum and Kwon’s “The Ecology of Withdrawal” published in Frontiers of Cultural Psychology in 2016. Varnum and Kwon’s “The Ecology of Withdrawal” was a commentary on “The *NEET* and *Hikikomori* (N/H) spectrum: Assessing the risks and consequences of becoming culturally marginalized” by YU and VN published in Frontiers in Cultural Psychology in 2015. YU is the second author of this rejoinder but was the first author to which Varnum and Kwon’s commentary was directed to, so she provided additional insights about what to include in this rejoinder, including ideas about the importance of conducting a multilevel analysis. KT is the third author of this rejoinder and has expertise on the evolutionary psychology perspective to which this rejoinder is responding to. KT has played an important role in making sure that the logic in this rejoinder challenges the logic put forth by Varnum and Kwon’s commentary. YU received the support from the grant #P15-1-05 (psychological well-being and mechanism on social interaction and coexistence) obtained from Kokoro Research Center, Kyoto University.

## Conflict of Interest Statement

The authors declare that the research was conducted in the absence of any commercial or financial relationships that could be construed as a potential conflict of interest.
